# The Relationship of Adherence to the Mediterranean Diet with Disease Activity and Quality of Life in Crohn’s Disease Patients

**DOI:** 10.3390/medicina60071106

**Published:** 2024-07-08

**Authors:** Athanasios Migdanis, Ioannis Migdanis, Nikoleta D. Gkogkou, Sousana K. Papadopoulou, Constantinos Giaginis, Athanasios Manouras, Maria Anna Polyzou Konsta, Rena I. Kosti, Konstantinos A. Oikonomou, Konstantinos Argyriou, Spyridon Potamianos, Andreas Kapsoritakis

**Affiliations:** 1 Nutrition and Dietetics Department, University of Thessaly, Argonafton 1C, 42132 Trikala, Greece; i.migdanis@aegeancollege.gr (I.M.); amanouras@uth.gr (A.M.); renakosti@uth.gr (R.I.K.); 2 Faculty of Medicine, University of Thessaly, Viopolis Mezourlo, 41110 Larissa, Greece; spotam@uth.gr (S.P.); kapsoritakis@uth.gr (A.K.); 3 MSc Program Nutrition in Health and Disease, Faculty of Medicine, University of Thessaly, Viopolis Mezourlo, 41110 Larissa, Greece; gogounikoleta11@gmail.com; 4 Department of Nutritional Sciences and Dietetics, International Hellenic University, Nea Moudania, 57001 Thessaloniki, Greece; sousana@the.ihu.gr; 5 Department of Food Science and Nutrition, University of the Aegean, Myrina, 81400 Lemnos, Greece; cgiaginis@aegean.gr; 6 Department of Nephrology, University Hospital of Larissa, Viopolis Mezourlo, 41110 Larissa, Greece; polyzoukonsta@yahoo.com; 7 Department of Gastroenterology, University Hospital of Larissa, Viopolis Mezourlo, 41110 Larissa, Greece; acoseko@yahoo.com (K.A.O.); kosnar2@yahoo.gr (K.A.)

**Keywords:** inflammatory bowel disease, mediterranean diet, disease activity, quality of life, Chron’s disease

## Abstract

*Background and Objectives*: Emerging evidence is placing the Mediterranean diet (MD) in the spotlight as a potential dietary model that could benefit inflammatory bowel disease (IBD) patients in terms of prevention and progress of the disease. The main aim of the present study is to shed some light on the relationship between the adherence to the MD and the degree of disease activity, as well as the quality of life in patients with Crohn’s disease (CD). *Materials and Methods*: An administered questionnaire was used to assess and record a number of parameters, including recent medical and weight history, anthropometric characteristics, disease activity (in remission or active disease), and quality of life of both male and female CD patients. Moreover, the level of compliance of the participants to the Mediterranean diet model was evaluated and its relationship with disease activity and quality of life was investigated. *Results*: Adherence to the MD was significantly higher in patients with inactive disease than in those with active disease (*p* = 0.019). According to the correlation analysis conducted, adherence to the MD was negatively correlated with disease activity (*p* = 0.039) and positively correlated with quality of life (QoL) (*p* = 0.046) of the participants. Intake of fruits, vegetables, and dairy products was significantly higher in remission patients (*p* = 0.046, *p* = 0.001, *p* = 0.041, respectively). *Conclusions*: We conclude, according to the findings of the study, that adherence to the MD is associated with disease activity and QoL in patients with CD. Future research should focus on MD intervention studies on IBD patients in order to assess its effect on modulating disease activity/course and related inflammatory biomarkers.

## 1. Introduction

The prevalence of inflammatory bowel diseases (IBDs), including Crohn’s disease (CD) and ulcerative colitis (UC), is on the increase [1]. Although the pathophysiology of IBDs is, to an extent, unknown, it is believed that they are of multifactorial etiology, where some environmental factors (such as stress, pollution, pathogen infections, smoking) act on a genetic background, resulting in persistent gastrointestinal tract inflammation [2]. Moreover, gut microbiota and immunological imbalance play a pivotal role in the pathogenesis [3]. Among environmental factors, diet is widely believed to play an important role in the progression of IBDs [4,5,6]. Despite the fact that the precise pathophysiological mechanisms are still unclear, several possible explanations have been suggested. Diet seems to play an important part in the equilibrium of gut microenvironment, having an effect on the gut’s microbial composition and functioning [4]. Furthermore, the nutritional patterns and habits followed by the developed Western countries, including high intake of red meat, refined carbohydrates, ultraprocessed and industrialized foods, high total fat, and especially saturated fat, foods, and low quantity of vegetables, fruit, and legumes, have been associated with increased mucosal inflammation and decreased amount of bacteria involved in fiber degradation [5,7].

In comparison to the Western-style diet, the Mediterranean diet (MD) is a more plant-based dietary model which consists of high amounts of vegetables and fruit (rich in antioxidants and vitamins), nuts, cereal, and extra virgin olive oil (rich in monounsaturated fatty acids), a moderate consumption of poultry, fish, and dairy products, and a low intake of processed red meat products and saturated fats [8]. The Mediterranean diet has gained recognition as a balanced beneficial nutritional model that lowers the risks of a variety of pathological states and human disorders and promotes human health [9]. Although the scientific literature supports a beneficial effect of Mediterranean diet on chronic conditions such as cardiovascular disease, metabolic disorders, some types of cancer, and neurological diseases [10,11,12], the influence of MD on IBDs remains unclear.

The exact mechanisms underpinning the association between MD and IBDs seem to be yet unspecified, although several potential hypotheses have been proposed. Research has shown that features of the Mediterranean diet, such as intake of vegetables, fruits, red wine, yogurt, nuts, and fish, have been associated with increased microbial diversity and benefits on gut microbiota in healthy individuals [13,14,15]. In studies conducted on patients with CD and UC, the results showed that Mediterranean diet interventions can significantly alter gut microbiota composition, increasing abundances of Faecalibacterium, Alistipes finegoldii, Ruminococcus bromii, and total short-chain fatty acid (SCFA) concentrations [16,17]. SCFAs such as acetate and butyrate can help maintain mucosal barrier function and modulate immune function in IBDs [18]. Other studies have also demonstrated that following an MD can have an effect in modulating the expression of inflammation associated genes and indicate a potential favorable anti-inflammatory effect [18,19]. The anti-inflammatory effects of the MD might also derive from the high intake of antioxidants such as polyphenols [20] and polyunsaturated fatty acids (omega 3/6) intake, which are believed to fuel the inflammatory process [21].

Although research in the existing literature has detected some beneficial effects of MD on gut microbiota and the mucosal inflammation process and has tried to assess the level of compliance of the MD in IBD patients in general, and more specifically between active and inactive disease phase, the scientific evidence on the effect of MD on clinical or laboratory disease activity and QoL of IBD patients is still very limited. The main aim of the present study is to provide some insight into the relationship between the adherence to the MD and the degree of disease activity, as well as the QoL in patients with CD, in a Greek population (a very understudied population).

## 2. Materials and Methods

This was a study with a cross-sectional design. The study was initiated in June 2020 and completed in February 2021. The study was conducted among 60 male and female patients diagnosed with CD. An administered questionnaire was used to assess and record a number of parameters, including recent medical and weight history, level of education, anthropometric characteristics, disease activity, and quality of life. Sample collection was carried out in the outpatient’s clinic of the Gastroenterology unit of General University Hospital of Larissa, the Hellenic Society of Crohn’s Disease and Ulcerative Colitis patients, and a private gastroenterology practice. The questionnaires were administered both in printed form and electronically. A registered dietitian was available during administration in case of needed clarifications. Participants who had a chronic or acute condition (e.g., food allergies, pancreatitis, had undergone major surgery, were undergoing chemo/radiotherapy, or had a recent infection) that might have had an impact on their recent nutritional state were excluded from the study. Patients were included if they were >15 years old and were diagnosed with CD. From all the patients that met the eligibility criteria for inclusion, 15 patients, 9 male and 6 female, refused to take part in the study.

More specifically, the disease activity of the participants was assessed with the Harvey-Bradshaw Index (HBI), a clinical tool for assessment of disease activity in patients with Crohn’s disease [22]. The tool consists of a few questions that allow the evaluation of CD’s degree of illness (severity) and detect remission. It has been developed to quantify the symptoms of the disease, including questions regarding general wellbeing, abdominal pain, stool consistency, and complications related to the disease (e.g., uveitis, arthralgia, abscess, etc.). In order to assess the quality of life (QoL) of the participants, the Crohn’s and ulcerative colitis questionnaire CUCQ-8 was used [23]. This is a shorter version of the more lengthy CUCQ-32 and is a validated tool to assess the QoL of patients with IBD. The CUCQ-8 questionnaire evaluates intestinal problems in the prior 2 weeks and their effects on QoL. It assesses loose or runny bowel movement, tiredness, frustration, urgency or awakening at night to use the toilet, and social inhibition. The total score is 0 to 24, and the higher the score, the lower the QoL. Several biochemical markers, including serum hemoglobin (gr/dL), hematocrit (%), C-reactive protein (CRP) (mg/L), albumin (g/dl), glucose (mg/dL), alkaline phosphatase (IU/L), glutamic–oxaloacetic transaminase SGOT (IU/L), glutamic–pyruvic transaminase SGPT (IU/L), γ-glutamyl transferase γ-GT (IU/L), amylase (IU/L), lactate dehydrogenase (U/L), vitamin B12 (pg/mL), triglycerides (mg/dL), folic acid (ng/mL), white blood cells (K/μL), and potassium (mmol/L), were also measured and quantified with an automatic biochemical analyzer in all participants. All patients that agreed to participate in the study were asked to visit the hospital’s outpatient clinic on a morning that was convenient for them, after overnight fasting, where blood sample collection by a registered nurse took place. Appetite and nutritional status of the participants was evaluated via nutrition risk screening 2002 (NRS-2002) [24] and simplified nutritional appetite questionnaire [25]. Physical activity of the participants was assessed using physical activity level (PAL) categories according to lifestyle [26].

The level of adherence of the participants to the Mediterranean diet model was evaluated using the Mediterranean diet score tool (MedDiet score) [27]. The MedDiet score is a tool created to assess the adherence of a population to the Mediterranean diet, which has been acknowledged worldwide as a model for a healthy and balanced diet. The MedDiet score tool particularly uses 11 main components of the Mediterranean diet (legumes, olive oil, poultry, red meat, nonrefined cereals, vegetables, fruits, fish, potatoes, dairy products, and alcohol). To evaluate the level of consumption of the above food groups, a 5-point scale is used, ranging from 0 = no consumption to 5 = daily consumption, respectively. Informed consent was obtained from all participants before entering the study. The trial protocol was approved by the Ethics Committee of the University of Thessaly (ethics approval code number 27624/6-4-2020), and adhered at all times to the Helsinki Declaration.

### Statistical Analysis

Statistical analysis was conducted using Statistical Product and Service Solutions, version 26 (IBM SPSS Statistics for Windows, Armonk, NY, USA). To determine the normality of the distribution of the examined variables, the Kolmogorov–Smirnov test was carried out. Continuous variables such as anthropometric characteristics and age are presented as means and ±standard deviations, and ordinal or categorical variables are presented as percentages (%). To assess possible differences in different measured parameters between active and inactive disease patients, chi-square test, independent samples t-test, and Mann–Whitney U-test were conducted. To examine possible correlations between MedDiet Score and disease activity or QoL, Pearson’s r and Spearman’s rho correlation coefficients were used. Statistical significance is reported as *p* < 0.05.

## 3. Results

A total of 60 participants consented and were recruited for the study. The mean age of the participants was 37.6 ± 12.5 (36.7% males and 63.3% females). The participants’ mean BMI was slightly above the normal range (25.1 ± 5.7 kg/m^2^), which falls within the overweight category. More specifically, 21.7% and 16.7% of the participants in the study were overweight and obese, respectively (Table 1). Regarding the disease activity according to HBI and QoL using the CUCQ-8, participants had a mean score of 5.65 ± 5.2 and 9.3 ± 5.8, respectively (Table 1). Furthermore, participants scored a mean of 30 ± 3.6 on the MedDiet Score, where the score ranges from 0 to 55, with higher values indicating greater adherence to the Mediterranean diet (Table 1).

According to HBI, 28 patients were in remission (score ≤ 4) and 32 had an active disease (score > 4) (Table 2). Statistical differences in age, QoL, adherence to the MD, and nutritional intake were observed between active and inactive disease patients. More specifically, patients with active disease were significantly older and had a significantly reduced dietary intake over the last week compared to inactive disease patients (*p* = 0.020 and *p* = 0.047). Moreover, MedDiet Score was significantly higher in patients with inactive disease than in those with active disease (*p* = 0.019). As hypothesized, patients in remission had a significantly lower CUCQ-8 score compared to active disease patients (*p* = 0.001) (Table 2). Concerning the biochemical markers measured, no statistical differences were observed in any of them between active and inactive disease patients (Table 3).

**Figure 1:**
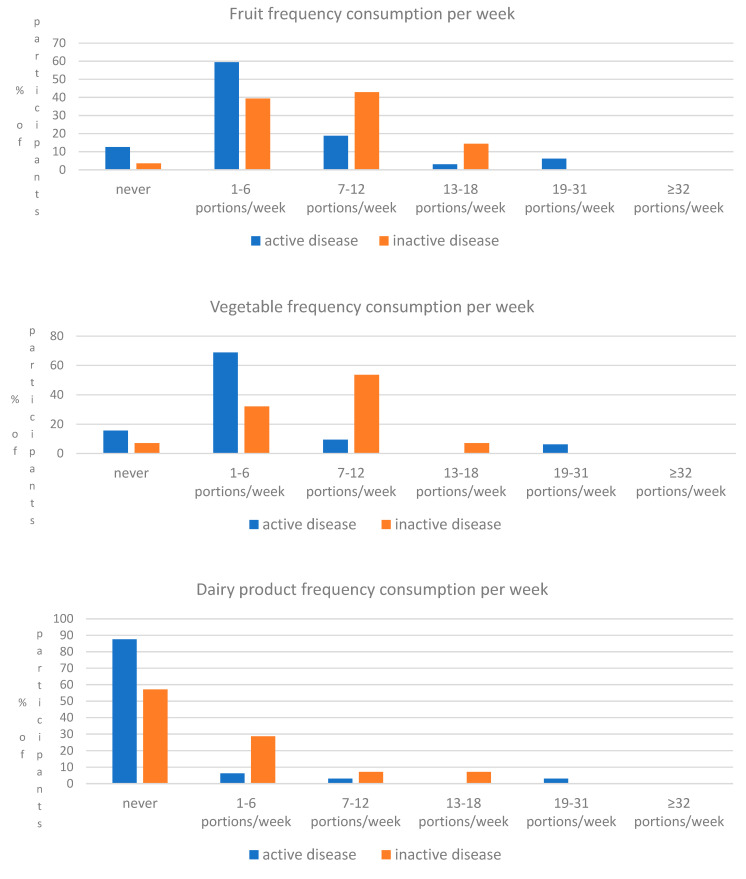
Intake of fruits, vegetables, and dairy products between active and inactive disease patients (*p* = 0.046, *p* = 0.001, *p* = 0.041, respectively).

According to the correlation analysis conducted, a significant negative correlation of the MedDiet Score with HBI (r = −0.267, *p* = 0.039) and CUCQ-8 (rho = −0.197, *p* = 0.046) was noted (Table 4). Lastly, it was observed that in some food groups used to assess the adherence of the participants to the Mediterranean diet, between active and inactive disease patients, statistical differences were observed (Table 5). More specifically, intake of fruits, vegetables, and dairy products was significantly higher in remission patients (*p* = 0.046, *p* = 0.001, *p* = 0.041, respectively) (Figure 1).

## 4. Discussion

The results of the study show that the mean BMI of the participants was above the normal range, with an overall percentage of 38.4% being overweight or obese. Although IBDs are traditionally associated with weight loss and low BMI (due to decreased food intake and malabsorption), this may be changing, and, paradoxically, overweight is now emerging as a further nutritional issue affecting people with CD [28,29,30]. The increasing prevalence of overweight and obesity in CD can be attributed to the westernized modern way of eating, sedentary lifestyle, and the medical treatment of IBDs, including the utilization of corticosteroids [31]. Moreover, some studies suggest that in patients with IBDs, imbalance of the gut microenvironment and altered metabolic intestinal signaling can have an impact on the development of obesity and dysmetabolism [32,33]. Furthermore, it is hypothesized that obesity can be a potential risk factor for the development of IBDs, possibly due to its overall proinflammatory effect [34,35]. Lastly, the literature supports that obesity may affect the disease course of treatment response of IBDs and that it might have a negative impact on a patient’s response to drug therapy, by affecting the pharmacokinetics of drugs [36,37].

The present study’s findings indicate that adherence to the MD was generally moderate in patients with CD. These findings seem to be in agreement with similar studies that have tried to assess the level of compliance of the MD to such group of patients. In a study from Australia that was conducted on 100 outpatients with IBDs, participants showed a low alignment with the Mediterranean diet characteristics [38]. According to the dietary assessment and the nutritional information obtained, participants consumed considerably fewer vegetables and grains than the Australian dietary recommendations. In another study that took place in Croatia, the authors focused on the dietary habits and attitudes of 44 patients with UC and 50 patients with CD [39]. Adherence to the MD was assessed using the Mediterranean Diet Service Score (MDSS), and the results showed low compliance of the subjects to the MD, with only nine participants fulfilling the criteria for Mediterranean diet adherence. In a different cross-sectional study by Taylor et al., analysis of the dietary intake of CD patients in remission again revealed poor alignment with the traditional Mediterranean diet characteristics [40]. It has been suggested that individuals with IBDs often avoid specific foods (e.g., spicy foods/meals, high-fiber foods) in order to manage symptoms [41,42], although there is no strong evidence that suggests that a reduction in dietary fiber is clinically necessary, with the exception of when fiber is found to be associated closely with a decline in gastrointestinal symptoms, or in Crohn’s disease patients with advanced luminal strictures [43].

With regard to differences between active and inactive disease patients, it was observed in the present study that MedDiet Score was significantly higher in patients with inactive disease than in those with active disease. According to the existing literature on the subject, a study from Italy produced similar results. In the Italian study by Fiorindi et al., adherence to the MD was assessed by a dietitian using the Medi-Lite questionnaire in 62 CD and 18 UC patients [44]. Among CD patients, adherence to the MD was higher in patients with inactive disease than in patients during the active phase, while no significant difference was found regarding disease activity in UC patients. In a similar study from Greece, where researchers emphasized the dietary assessment of CD patients, analysis of the results identified a higher compliance to the MD in patients with inactive CD [45]. The tool used to evaluate adherence to the MD was also the MedDiet Scoring method, which, due to its scoring large scale, is considered more informative when compared with smaller-scale scoring tools in terms of capturing a nutritional behavior or pattern [27]. As mentioned above, patients with IBDs often have the tendency to avoid a variety of foods in an attempt to prevent/relieve digestive symptoms (abdominal pain/bloating, diarrhea, frequent and/or urgent bowel movements, cramping, etc.) or relapse. A study published by Casanova et al. noted that 86% of patients with active disease move onto stricter diets, while 77% of patients avoid certain foods during remission [46].

Another finding of the present study that is worth mentioning is the fact that adherence to the MD was negatively correlated with disease activity and positively correlated with QoL of the participants. Current evidence coming to the surface indicates that adherence to a traditional Mediterranean dietary model may reduce disease activity in individuals with IBD. In a study by Chicco et al., significantly fewer patients with CD and UC who followed a dietary intervention (traditional Mediterranean diet) for six months had active disease [47]. Furthermore, compliance to the MD was associated with significant improvement of disease activity, inflammatory biomarkers (C-reactive protein and fecal calprotectin), and quality of life. In another study that took place in Turkey, the authors concluded that stronger adherence to the MD in patients with ulcerative colitis can help improve quality of life and modulate disease activity [48]. In this study, the Mediterranean Diet Adherence Scale was used to evaluate adherence to the Mediterranean diet, Crohn’s Disease Activity Index was used to evaluate disease activity in Crohn’s disease, and disease activity was determined by using the Mayo Clinic score for ulcerative colitis. Additionally, in the previously mentioned Greek study by Papada et al., the MedDiet score correlated positively with quality of life assessment and negatively with disease activity, assessed via the Harvey-Bradshaw Index score and CRP [45]. In an Egyptian randomized controlled trial (RCT) that included pediatric patients with mild to moderate active CD and UC, the group randomized to receive the MD intervention demonstrated a greater reduction in clinical scores, fecal calprotectin, and inflammatory cytokines [49]. Similarly, in another pediatric patient study, adherence to the MD was associated with decreased fecal calprotectin levels in children with CD under biological therapy [50]. Interestingly, in an intervention study that was performed on adults with CD, MD was well tolerated and associated with symptomatic remission and improvement in QoL measures [51]. The potential mechanisms through which MD can have a beneficial effect on the course of IBDs are its impact on gut microbiome composition [52,53] and its anti-inflammatory effects [54,55]. To date, there are no current international dietary recommendations for IBD patients. According to ESPEN guidelines, there is no oral diet that can be generally recommended to promote remission in IBD patients with active disease, or to obtain remission in remission phases [56]. The only nutritional approach with strong evidence of efficacy in inducing remission is exclusive enteral nutrition (EEN), a diet based on liquid nutrition formulas delivered enterally (either orally or via nasogastric tube) [56,57]. In some cases, EEN has had comparable efficacy to steroid treatment in inducing remission in children and adults with CD but significantly fewer side effects [58,59].

Lastly, it was observed in the present study that in some food groups used to assess the adherence of the participants to the Mediterranean diet, between active and inactive disease patients, statistical differences were observed. More specifically, intake of fruits, vegetables, and dairy products was significantly higher in remission patients. These results are similar to those reported in other studies in the literature. In the study by Fiorindi et al., results showed significant differences in daily consumption of high-fiber foods (fruits, vegetables, and legumes) in relation to disease activity in both CD and UC patients [44]. In the study by Marsh et al., the study cohort (41 CD and 51 UC outpatients) did not meet the MD targets for extra virgin olive oil, vegetables, legumes, and fruit [38]. The studies from Croatia and Canada observed similar findings, where the IBD population that participated had a poor adherence rate to food groups that are a hallmark for the MD, such as fresh fruit and vegetables [39,40]. Generally, patients with IBD tend to reduce the consumption of a variety of foods, particularly spicy foods, alcohol, dairy products, high-fiber foods, fruits, and vegetables, especially during disease flare-ups. This is probably attributed to the effect of fiber, as in some cases it is believed to exacerbate gastrointestinal symptoms in patients with IBDs [60]. On the other hand, in a large prospective cohort, CD patients who reported that they did not avoid high-fiber foods were significantly less likely to have a disease flare-up [61]. Fiber-degrading bacteria and their concentration in individuals’ gut microbiota seems to differentiate the effects of dietary fiber on IBD patients [6,62].

Scientific evidence to support the effect of other dietary regimens, including the paleolithic diet, gluten-free, low-FODMAP (fermentable oligo-, di-, monosaccharides, and polyols), specific carbohydrate, or ω-3 PUFA enriched diets, on intestinal inflammation or on inducing remission in IBD patients is presently lacking [56,63]. Moreover, avoidance of specific foods and strict diet plans are associated with malnutrition and micronutrient deficiencies [46]. It seems as if the role of nutrition in the management of IBDs has been so far underestimated. In light of recent findings, it seems that MD is emerging as one of the most perspective dietary choices for IBD patients.

Limitations of the study include restricted sample size and an observational design which can potentially affect the results’ external validity and does not allow us to determine the causality of the associations, but our findings can add information/evidence to the existing literature and suggest directions for future research with larger sample sizes and a nutritional intervention design. Also, the tools used to assess disease activity and quality of life are well accepted and valid, but are simpler versions of the Crohn’s Disease Activity Index (CDAI) and CUCQ-32, respectively, which are lengthier tools and can assess further parameters; however, they were chosen for data collection purposes. Lastly, the relatively high level of drop-out can be attributed to the fact that sample collection took place during the COVID-19 pandemic period and this might have also introduced selection bias since many patients were skeptical about visiting the hospital’s outpatient clinic at that period. Sampling bias can be a major threat to research validity during the COVID-19 pandemic. This would result in studies with samples that are less representative of the target population, risking the generalizability of the research [64]. 

## 5. Conclusions

We conclude, according to the results of the study, that adherence to the MD is associated with disease activity and QoL in patients with CD. Future research should focus on MD intervention studies on IBD patients in order to assess its effect on modulating disease activity/course and related inflammatory biomarkers. Personalized nutritional assessment and nutritional counseling/education regarding MD and generally balanced nutrition might need to be included in the routine clinical management of IBD patients in order to optimize their overall food-related quality of life, prevent malnutrition, and alleviate related symptoms.

## Figures and Tables

**Table 1:** Demographic, anthropometric, nutritional, and clinical characteristics of the participants.

Variables	Number of Participants (%)/Mean ± SD
Gender Males Females	22 (36.7%) 38 (63.3%)
Weight (kg)	80.4 ± 15.5
BMI (kg/ m^2^) <18.5 (kg/ m^2^) 18.5–25 (kg/ m^2^) 25–30 (kg/ m^2^) >30 (kg/ m^2^)	25.1 ± 5.7 4 (6.7%) 33 (55%) 13 (21.7%) 10 (16.7%)
Age (years)	37.6 ± 12.5
Smoking status ex-smoker smoker at present never smoked	25 (41.7%) 16 (26.7%) 35 (58.3%)
Pharmacological therapy aminosalicylates antibiotics corticosteroids immunosuppressants biologics surgical therapy	22 (36.7%) 3 (5%) 2 (3.3%) 11 (18.3%) 22 (36.7%) 16 (26.7%)
Physical activity level Never Rarely Often High	16 (26.7%) 29 (48.3%) 7 (11.7%) 8 (13.3%)
Appetite poor moderate good very good	1 (1.7%) 15 (25%) 23 (38.3%) 21 (35%)
Number of meals per day one per day two per day three per day more than three per day	5 (8.3%) 13 (21.7%) 22 (36.7%) 20 (33.3%)
Harvey-Bradshaw Index (HBI)	5.65 ± 5.2
CUCQ-8	9.3 ± 5.8
MedDiet Score	30 ± 3.6

BMI, body mass index; SD, standard deviation.

**Table 2:** Comparison in different variables between patients with active and inactive disease.

Variables	Active (*n* = 32) (%/Mean ± SD)	Inactive (*n* = 28) (%/Mean ± SD)	*p* Value
Gender Male Female	9 (28.1%) 23 (71.9%)	13 (46.4%) 15 (53.6%)	0.142
Age years	41 ± 11	33 ± 12	0.020
BMI (kg/m^2^)	26.4 ± 6	23.6 ± 4	0.063
Smoking status smoker at present nonsmoker	16 (34.4%) 21 (65.6%)	8 (17.9%) 23 (82.1%)	0.149
Physical activity level Never Rarely Often High	9 (28.1%) 19 (59.4%) 2 (6.2) 2 (6.2%)	7 (25%) 10 (35.7%) 5 (17.9%) 6 (21.4%)	0.107
CUCQ-8	12.6 ± 5	5.5 ± 3	0.001
MedDiet Score	29 ± 3	31.2 ± 3	0.019
Appetite poor moderate good very good	1 (3.1%) 9 (28.1%) 12 (37.5%) 10 (31.2%)	0 (0%) 6 (21.4%) 11 (39.3%) 11 (39.3%)	0.698
Reduced dietary intake (last week) No Yes	25 (78.1%) 7 (21.9%)	26 (92.9%) 2 (7.1%)	0.047

BMI, body mass index; SD, standard deviation; CUCQ-8, Crohn’s and ulcerative colitis questionnaire-8.

**Table 3:** Biochemical indices between inactive and active disease patients.

	Active (*n* = 32) (mean ± SD)	Inactive (*n* = 28) (mean ± SD)	*p* Value
Hemoglobin (gr/dL)	12.8 ± 1.7	12.9 ± 1.3	0.817
Hematocrit (%)	38.7 ± 4	39.4 ± 4	0.577
CRP (mg/L)	3.5 ± 4	2 ± 3	0.265
Albumin (g/dL)	4.3 ± 0.4	4.5 ± 0.6	0.481
Glucose (mg/dL)	91 ± 11	90 ± 8	0.875
Alkaline phosphatase (IU/L)	70 ± 46	85 ± 41	0.347
SGOT (IU/L)	19.7 ± 10	18.7 ± 8	0.775
SGPT (IU/L)	24.5 ± 22	18.3 ± 11	0.315
γ-GT (IU/L)	34 ± 42	18 ± 11	0.140
Amylase (IU/L)	64 ± 20	70 ± 28	0.645
Lactate dehydrogenase (U/L)	241 ± 155	174 ± 71	0.327
B12 (pg/mL)	482 ± 304	419 ± 203	0.505
Triglycerides (mg/dL)	127 ± 66	90 ± 55	0.276
Folic acid (ng/mL)	10.6 ± 6	11.9 ± 6	0.661
White Blood Cells (K/μL)	7.6 ± 2	7.1 ± 2	0.466
Potassium (mmol/l)	4.3 ± 0.3	4.3 ± 0.3	0.761

SD, standard deviation; SGOT, glutamic–oxaloacetic transaminase; SGPT, glutamic–pyruvic transaminase; γ-GT, γ-glutamyl transferase; CRP, C-reactive protein.

**Table 4:** Correlation analysis between the MedDiet Score and disease activity and QoL.

	r/rho	*p* Value
HBI	−0.267	0.039
CUCQ-8	−0.197	0.046

HBI, Harvey-Bradshaw Index; CUCQ-8, Crohn’s and ulcerative colitis questionnaire-8.

**Table 5:** Food groups of MedDiet Score according to disease activity.

Food Groups	Frequency	Active (*n* = 32) (Number of Participants/%)	Inactive (*n* = 28) (Number of Participants/%)	*p* Value
Nonrefined cereals	Never 1–6 portions/week 7–12 portions/week 13–18 portions/week 19–31 portions/week ≥32 portions/week	12 (37.5%) 15 (46.9%) 5 (15.6%) 0 (0%) 0 (0%) 0 (0%)	9 (32.1%) 11 (39.3%) 7 (25%) 0 (0%) 1 (3.6%) 0 (0%)	0.548
Potatoes	Never <1 portion/week 1–2 portions/week 3 portions/week 4 portions/week >4 portions/week	0 (0%) 9 (28.1%) 11 (34.4%) 6 (18.8%) 4 (12.5%) 2 (6.2%)	1 (3.6%) 1 (3.6%) 15 (53.6%) 5 (17.9%) 4 (14.3%) 2 (7.1%)	0.163
Fruits	Never 1–4 portions/week 5–8 portions/week 9–15 portions/week 16–21 portions/week ≥22 portions/week	4 (12.5%) 19 (59.4%) 6 (18.8%) 1 (3.1%) 2 (6.2%) 0 (0%)	1 (3.6%) 11 (39.3%) 12 (42.9%) 4 (14.3%) 0 (0%) 0 (0%)	0.046
Vegetables	Never 1–6 portions/week 7–12 portions/week 13–20 portions/week 21–32 portions/week ≥33 portions/week	5 (15.6%) 22 (68.8%) 3 (9.4%) 0 (0%) 2 (6.2%) 0 (0%)	2 (7.1%) 9 (32.1%) 15 (53.6%) 2 (7.1%) 0 (0%) 0 (0%)	0.001
Legumes	Never <1 portions/week 1–2 portions/week 3–4 portions/week 5–6 portions/week ≥6 portions/week	13 (40.6%) 11 (34.4%) 8 (25%) 0 (0%) 0 (0%) 0 (0%)	8 (28.6%) 4 (14.3%) 15 (53.6%) 1 (3.6%) 0 (0%) 0 (0%)	0.061
Fish	Never <1 portions/week 1–2 portions/week 3–4 portions/week 5–6 portions/week ≥6 portions/week	1 (3.1%) 12 (37.5%) 14 (43.8%) 3 (9.4%) 1 (3.1%) 1 (3.1%)	2 (7.1%) 8 (28.6%) 17 (60.7%) 1 (3.6%) 0 (0%) 0 (0%)	0.524
Red meat	≤1 portion/week 2–3 portions/week 4–5 portions/week 6–7 portions/week 8–10 portions/week >10 portions/week	9 (28.1%) 12 (37.5%) 9 (28.1%) 1 (3.1%) 1 (3.1%) 0 (0%)	9 (32.1%) 12 (42.9%) 2 (7.1%) 4 (14.3%) 1 (3.6%) 0 (0%)	0.198
Poultry	≤3 portions/week 4–5 portions/week 5–6 portions/week 7–8 portions/week 9–10 portions/week >10 portions/week	18 (56.2%) 10 (31.2%) 3 (9.4%) 1 (3.1%) 0 (0%) 0 (0%)	16 (57.1%) 6 (21.4%) 5 (17.9%) 1 (3.6%) 0 (0%) 0 (0%)	0.716
Dairy products	≤10 portions/week 11–15 portions/week 16–20 portions/week 21–28 portions/week 29–30 portions/week >30 portions/week	28 (87.5%) 2 (6.2%) 1 (3.1%) 0 (0%) 1 (3.1%) 0 (0%)	16 (57.1%) 8 (28.6%) 2 (7.1%) 2 (7.1%) 0 (0%) 0 (0%)	0.041
Olive oil	Never Rarely <1 portions/week 1–3 portions/week 3–5 portions/week everyday	0 (0%) 1 (3.1%) 2 (6.2%) 2 (6.2%) 2 (6.2%) 25 (78.1%)	0 (0%) 1 (3.6%) 0 (0%) 5 (17.9%) 0 (0%) 22 (78.6%)	0.264
Alcohol	<300 mL/day 300 mL/day 400 mL/day 500 mL/day 600 mL/day >700 or 0 mL/day	17 (53.1%) 3 (9.4%) 2 (6.2%) 1 (3.1%) 0 (0%) 9 (28.1%)	19 (67.9%) 5 (17.9%) 2 (7.1%) 0 (0%) 0 (0%) 2 (7.1%)	0.213

## Data Availability

The data generated and analyzed for this study can be found within the article and from the corresponding author on request.
